# Ag^+^ as a More Effective Elicitor for Production of Tanshinones than Phenolic Acids in *Salvia miltiorrhiza* Hairy Roots

**DOI:** 10.3390/molecules20010309

**Published:** 2014-12-24

**Authors:** Bingcong Xing, Dongfeng Yang, Wanli Guo, Zongsuo Liang, Xijun Yan, Yonghong Zhu, Yan Liu

**Affiliations:** 1 College of Life Science, Zhejiang Sci-Tech University, 928 Second Avenue, Xiasha Higher Education Zone, Hangzhou 310018, Zhejiang, China; E-Mails: xingbingcong@163.com (B.X.); yangdongfeng@zstu.edu.cn (D.Y.); gwl1016@aliyun.com (W.G.); 2 Tasly R&D Institute, Tasly Holding Group Co. Ltd, Tianjin 300410, China; E-Mails: yxj@tasly.com (X.Y.); zyh@tasly.com (Y.Z.); lyzc596392@163.com (Y.L.)

**Keywords:** *Salvia miltiorrhiza* Bunge, hairy roots, Ag^+^, phenolic acids, tanshinones

## Abstract

Phenolic acids and tanshinones are two groups of bioactive ingredients in *Salvia miltiorrhiza* Bunge. As a heavy metal elicitor, it has been reported that Ag^+^ can induce accumulations of both phenolic acids and tanshinones in *S. miltiorrhiza* hairy roots. In this study, the effects of Ag^+^ treatment on accumulations of six phenolic acids and four tanshinones in *S. miltiorrhiza* hairy roots were investigated. To further elucidate the molecular mechanism, expressions of key genes involved in the biosynthesis of these ingredients were also detected. The results showed that although the total phenolic acids content was almost not affected by Ag^+^, accumulations of rosmarinic acid (RA), caffeic acid and ferulic acid were significantly increased, while accumulations of salvianolic acid B (LAB), danshensu (DSU) and cinnamic acid were decreased. We speculate that LAB probably derived from the branch pathway of DSU biosynthesis. Contents of four tanshinones were enhanced by Ag^+^ and their accumulations were more sensitive to Ag^+^ than phenolic acids. Genes in the upstream biosynthetic pathways of these ingredients responded to Ag^+^ earlier than those in the downstream biosynthetic pathways. Ag^+^ probably induced the whole pathways, upregulated gene expressions from the upstream pathways to the downstream pathways, and finally resulted in the enhancement of ingredient production. Compared with phenolic acids, tanshinone production was more sensitive to Ag^+^ treatments. This study will help us understand how secondary metabolism in *S. miltiorrhiza* responds to elicitors and provide a reference for the improvement of the production of targeted compounds in the near future.

## 1. Introduction

**Figure 1:**
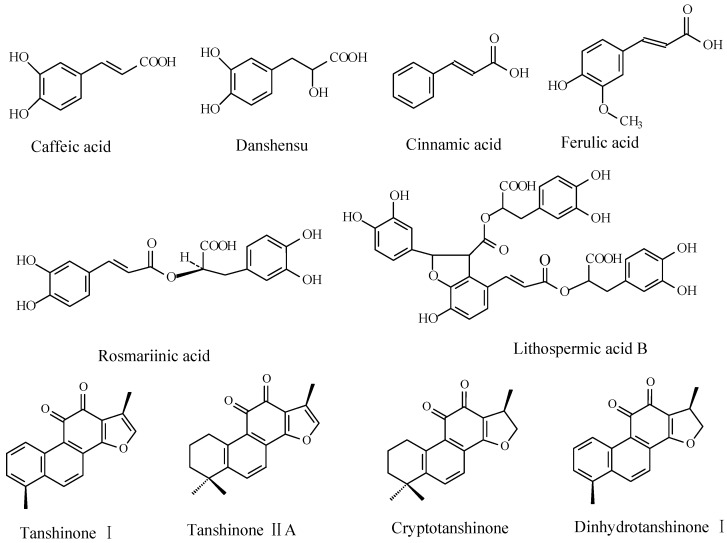
The chemical structure of ten compounds in *S. miltiorrhiza* have been studied in this article. Six water-soluble phenolic acids and four lipid-soluble tanshinones.

Salvia miltiorrhiza Bunge, known as “Dan Shen” in Chinese, is famous for the prevention and treatment of coronary heart disease. It contains two major groups of bioactive ingredients, namely phenolic acids and tanshinones [1]. The phenolic acids mainly include (R)-2-Hydroxy-3-(3,4-dihydroxyphenyl) propionic acid (danshensu, DSU), caffeic acid, cinnamic acid, ferulic acid, protocatechuic aldehyde, rosemarinic acid (RA) and salvianolic acid B (LAB) [2] and the tanshinones include tanshinone I (T-I), dihydrotanshinone I (DT-I), cryptotanshinone (CT), tanshinone II A (T-IIA) and tanshinone IIB (T-IIB) [3] (Figure 1). Phenolic acids have been widely used because of their potential in the treatment of atherogenic dyslipidemia and cholestatic liver injury [4]. Tanshinones are reported to have anti-platelet, cardioprotective, and anti-inflammatory effects [5,6,7].

**Figure 2:**
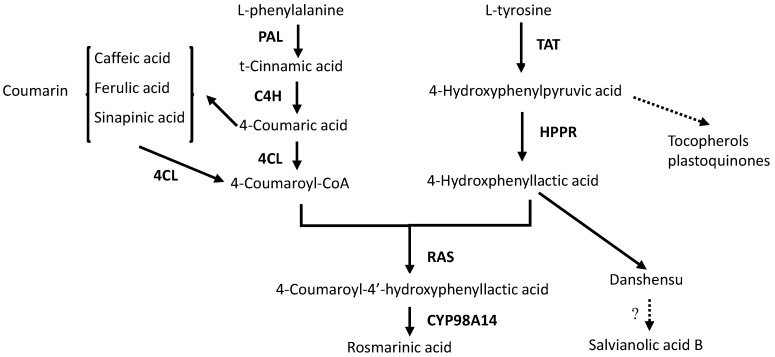
The metabolic pathways of rosmarinic acid and salvianolic acid B in *S. miltiorrhiza* hairy roots. Multiple enzymatic steps are represented by dotted lines. C4H, cinnamic acid 4-hydroxylase; 4CL, hydroxycinnamate coenzyme A ligase; HPPR, 4-hydroxyphenylpyruvate reductase; PAL, phenylalanine ammonia-lyase; RAS, rosmarinic acid synthase; TAT, tyrosine aminotransferase; CYP98A14, acytochrome P450-dependent monooxygenase.

Phenolic acids are mainly produced from the phenylpropanoid and tyrosine-derived pathways [8] (Figure 2). The biosynthetic pathways of rosmarinic acid have been elucidated. Phenylalanine ammonia-lyase (PAL), cinnamic acid 4-hydroxylase (C4H) and hydroxycinnamate coenzyme A ligase (4CL) are key genes in the phenylpropanoid pathway. Tyrosine aminotransferase (TAT) and 4-hydroxyphenylpyruvate reductase (HPPR) are two key genes in the tyrosine-derived pathway. Rosmarinic acid synthase (RAS) and CYP98A14 are two important enzymes that convert 4-coumaroyl-CoA and 4-hydroxphenyllactic acid to RA [9].

Phenolic acids are important effective compounds in *S. miltiorrhiza*, and their biosynthetic regulation has been widely studied. Many elicitors (biotic and abiotic) have been used to enhance phenolic acid production, such as polysaccharides, glycoproteins, low-molecular weight organic acids, fungal cell-wall materials, ultraviolet irradiation and heavy metal salts [8,10,11,12]. Our studies showed that salicylic acid (SA), abscisic acid (ABA), gibberellinic acid (GA), yeast extract (YE), methyl jasmonate (MeJA) and fungi extract could induce phenolic acid accumulation [8,13,14]. Overexpression of key genes in the biosynthetic pathway could promote secondary metabolite production [15]. Overexpression of key genes in LAB biosynthesis pathways (*PAL*, *C4H*, *4CL*, *TAT*, *HPPR* and *RAS*) could increase accumulation of phenolic acids, and the downregulation of these genes could reduce their accumulation in *S. miltiorrhiza* [16].

Terpenoids are biosynthesized via two pathways in plants: the mevalonate (MVA) pathway in the cytosol and the methylerythritol phosphate (MEP) pathway in plastids [7,17,18]. 3-Hydroxy-3-methylglutaryl CoA reductase (HMGR) is a rate-limiting enzyme and catalyzes formation of mevalonate (MVA) from 3-hydroxy-3-methylglutaryl CoA. 1-deoxy-D-xylulose 5-phosphate synthase (DXS) and 1-deoxy-D-xylulose 5-phosphate reductoisomerase (DXR) are the first two key enzymes in the MEP pathway. Geranylgeranyl diphosphate synthase (GGPPS) catalyzes the consecutive condensation of the dimethylallyl diphosphate (DMAPP) with three molecules of IPP to produce geranylgeranyl diphosphate (GGPP), the universal precursor for the biosynthesis of diterpenoids such as the tanshinones. GGPPS has been considered an important regulatory target in the tanshinone biosynthetic pathway [19]. GGPP is then converted by copalyl diphosphate synthase (CPS) and kaurene synthase-like (KSL) to miltiradiene, which has recently been identified as a precursor of tanshinone biosynthesis [20]. Elicitors such as La, MeJA, YE, SA and Ag^+^ can stimulate tanshinone accumulation in *S. miltiorrhiza* hairy roots [21,22,23]. Although expressions of *HMGR*, *DXR*, *DXS*, *GGPPS*, *CPS* and *KSL* can be induced by MeJA, we found that the MEP pathway probably plays a more important role in tanshinone accumulation [18]. Overexpression of *GGPPS*, *HMGR* and *DXS* in transgenic hairy root lines can significantly enhance tanshinone production [24]. RNA interference (RNAi) of *CPS* reduced dihydrotanshinone I and cryptotanshinone accumulations [20].

As a group of efficient abiotic elicitors, the use of metal ions including Ca^2+^, Co^2+^, Ag^+^, Cd^2+^, Cu^2+^, Ce^3+^, La^+^, Mn^2+^ and Zn^2+^ to induce bioactive ingredient production in plants has been widely researched [21,25,26]. Among them, Ag^+^ was the mostly studied. In *Vitis vinifera* cell suspension cultures, Ag^+^ at a low concentration (5.0 μM) stimulated 3-O-glucosylresveratrol production, but it did not affect cell growth [26]. High concentrations of Ag^+^ (30 μM) can enhance paclitaxel yield in suspension culture of *Taxus spp.* Cells [27]. Ag^+^ can also improve echinacoside and acteoside production in cell suspension cultures of *Cistanchede serticola* [28]. In *S. miltiorrhiza* hairy roots, treatment with 15 μM Ag^+^ significantly increased RA accumulation, as well as total phenolic acid content [12]. However, Xiao reported that LAB is more sensitive to Ag^+^ than RA, and the content of RA was not affected [29]. In terms of tanshinone accumulation, the previous work showed that tanshinone production was significantly enhanced by Ag^+^ probably via the non-MVA pathway [30]. There is currently a lack of information regarding the comparison of how the two groups of bioactive compounds in *S. miltiorrhiza* respond to Ag^+^.

In this study, to reveal the effects of Ag^+^ treatments on both phenolic acid and tanshinone production, and elucidate the regulatory mechanisms of the biosynthesis of these ingredients, accumulations of six phenolic compounds and four tanshinone compounds were examined. Simultaneously, expressions of 15 genes involved in biosynthesis of these ingredients were investigated.

## 2. Results and Discussion

### 2.1. Effects of Ag^+^ Elicitor on Cell Growth of S. miltiorrhiza Hairy Roots

**Figure 3:**
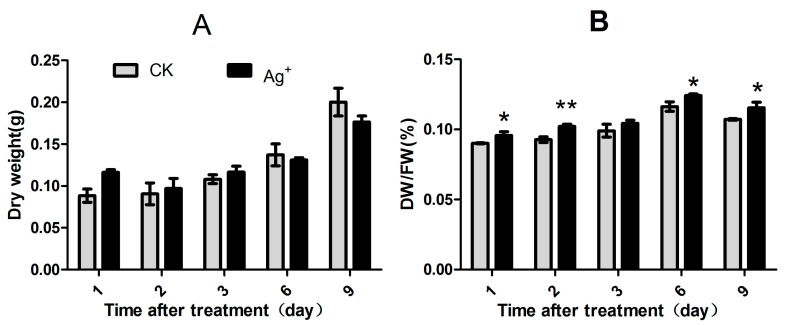
Effects of Ag^+^ elicitor on cell growth of *S. miltiorrhiza* hairy roots. (**A**) Dry weight (DW) of the *S. miltiorrhiza* hairy roots after Ag^+^ treatment; (**B**) Dry weight/Fresh weight (FW) ratio of the *S. miltiorrhiza* hairy roots after Ag^+^ treatment. The vertical bars show the SD values (*n* = 3). The asterisks indicate statistically significant differences at *p* < 0.05 (*, 0.01 < *p* < 0.05; **, 0.001 < *p* < 0.01.) between the content in the elicitor treated cultures and that in the corresponding controls.

Treatments with Ag^+^, the biomass of *S. miltiorrhiza* hairy roots in shake-flask cultures showed an increasing trend in both the control and the treatment groups (Figure 3A). Compared with the control, the hairy roots growth was almost not affected by Ag^+^ in the first 6 days after treatment and was slightly depressed on day 9. However, the dry weight (DW)/fresh weight (FW) was significantly increased at 1, 2, 6 and 9 days after treatment. This means that Ag^+^ elicitor decreased the growth of *S. miltiorrhiza* hairy roots and reduced the water content in hairy roots (Figure 3B).

### 2.2. Effects of Ag^+^ Elicitor on Accumulation of Phenolic Acids in S. miltiorrhiza Hairy Roots

In this study, six phenolic acids compounds were examined (RA, LAB, DSU, caffeic acid, ferulic acid and cinnamic acid).

**Figure 4:**
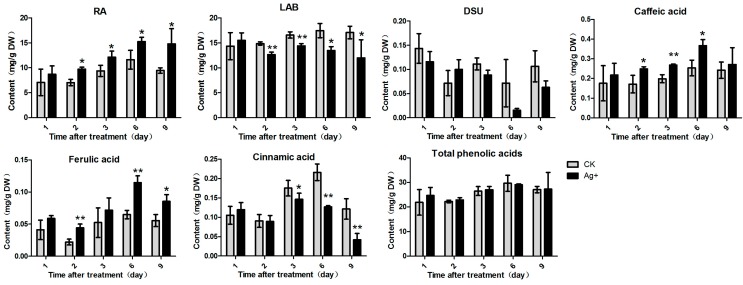
Effects of Ag+ on accumulations of phenolic acids in *S. miltiorrhiza* hairy roots. Content of RA (rosmarinic acid), LAB (salvianolic acid B), DSU, caffeic acid, ferulic acid, cinnamic acid and total phenolic acids. The vertical bars show the SD values (*n* = 3). The asterisks indicate statistically significant differences at *p* < 0.05 (*, 0.01 < *p* < 0.05; **, 0.001 < *p* < 0.01.) between the content in the elicitor treated cultures and that in the corresponding controls.

With Ag^+^ treatment, RA content was significantly increased to 15.27 mg/g DW on day 6 after treatment (1.3-fold of the control). Accumulations of caffeic acid and ferulic acid were also stimulated by Ag^+^, and the maximum contents were 0.37 mg/g DW and 0.11 mg/g DW, respectively. However, contents of DSU, cinnamic acid and LAB were significantly inhibited by Ag^+^, and were just 22%, 65% and 70% of the control levels, respectively. The results indicated that Ag^+^ induced accumulations of RA, caffeic acid and ferulic acid, but decreased contents of LAB, DSU and cinnamic acid. However, accumulation of the total phenolic acids was almost not affected by Ag^+^ treatment (Figure 4).

### 2.3. Effects of Ag^+^ Elicitor on Tanshinones Accumulation in S. miltiorrhiza Hairy Roots

**Figure 5:**
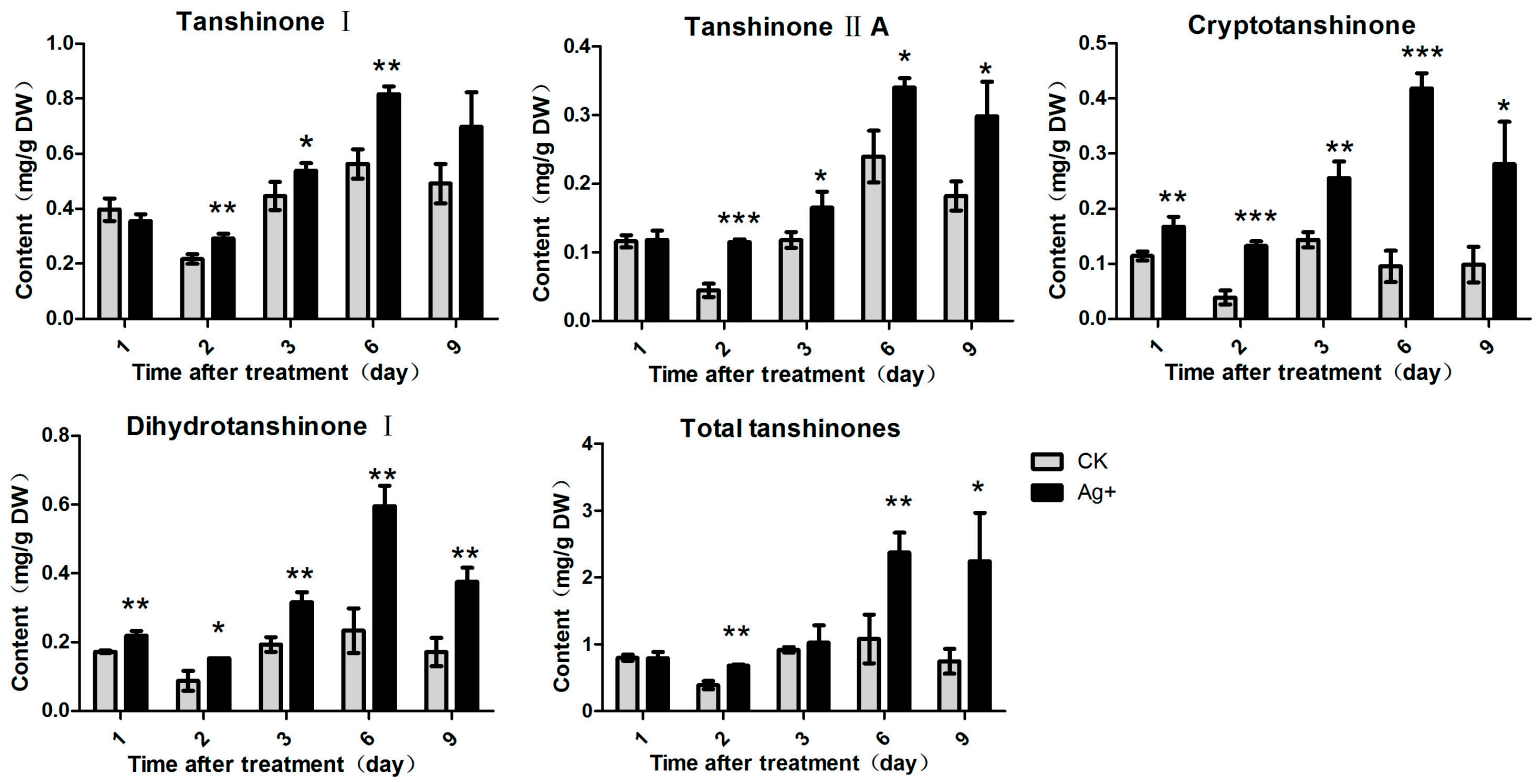
Effects of Ag^+^ on accumulations of tanshinones in *S. miltiorrhiza* hairy roots. Content of T-I (tanshinone I), T-IIA (tanshinone IIA), CT (cryptotanshinone), DT-I (dihydrotanshinone I) and total tanshinones. The vertical bars show the SD values (*n* = 3). The asterisks indicate statistically significant differences at *p* < 0.05 (*, 0.01 < *p* <0.05; **, 0.001 < *p* < 0.01; ***, *p* < 0.001.) between the content in the elicitor treated cultures and that in the corresponding controls.

Contents of four lipid-soluble tanshinones components (T-I, T-IIA, CT and DT-I) were examined in this study. The results showed that the four tanshinone components were induced by Ag^+^ and their contents reached a maximum on day 6 post-treatment (Figure 5). Contents of T-I, T-IIA, CT and DT-I reached to 0.82 mg/g DW (1.46-fold of the control), 0.34 mg/g DW (1.42-fold of the control), 0.42 mg/g DW (4.4-fold of the control) and 0.60 mg/g DW (2.56-fold of the control), respectively. The total tanshinone accumulation was significantly induced by Ag^+^. The maximum content of total tanshinones reached to 2.37 mg/g DW on day 6 post-treatment (2.2-fold of the control) (Figure 5).

### 2.4. Effects of Ag^+^ Elicitor on Genes Expressions Involved in the Two Parallel Pathways of LAB Biosynthesis

**Figure 6:**
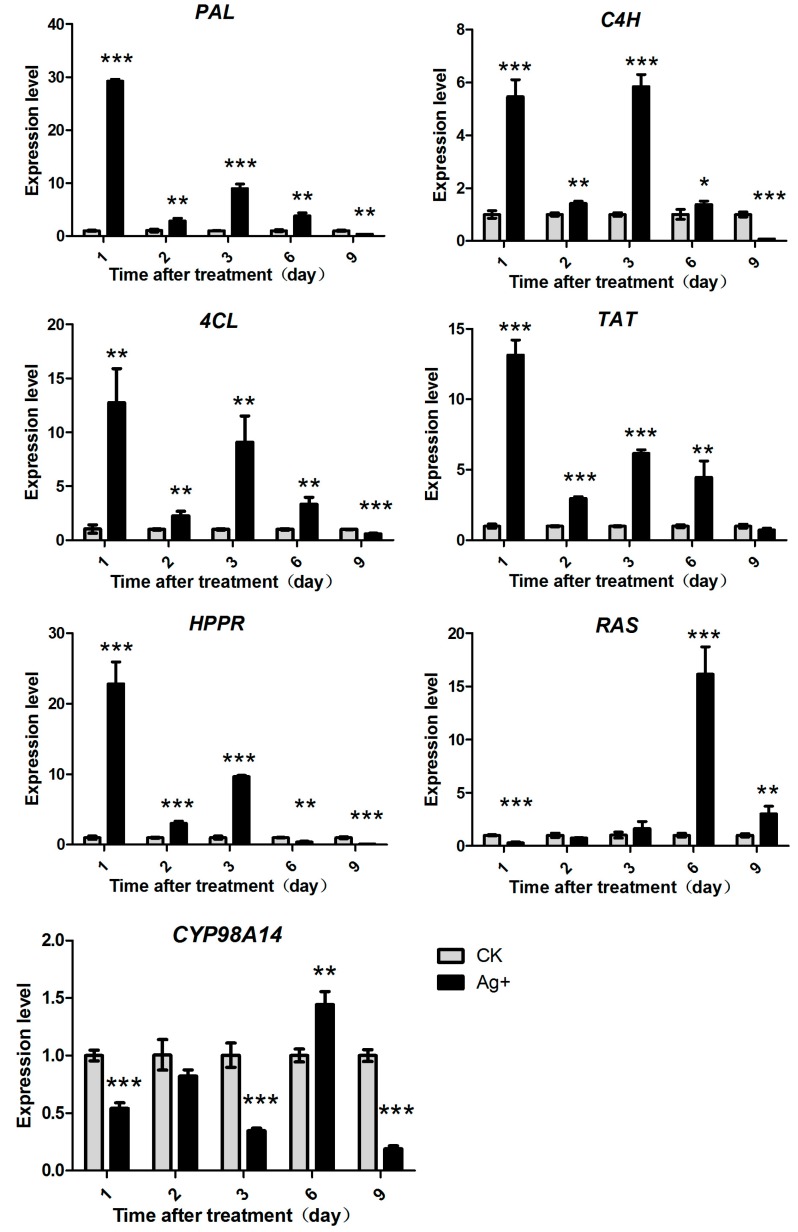
Effects of Ag^+^ on the expression level of seven key enzyme genes of RA biosynthesis pathway (phenylpropanoid pathway and the tyrosine pathway) in *S. miltiorrhiza* hairy roots. The vertical bars show the SD values (*n* = 3). The asterisks indicate statistically significant differences at *p* < 0.05 (*, 0.01 < *p* <0.05; **, 0.001 < *p* < 0.01; ***, *p* < 0.001.) between the content in the elicitor treated cultures and that in the corresponding controls.

Expressions of seven key genes in RA biosynthesis pathways were determined in this study, including *PAL*, *C4H*, *4CL*, *TAT*, *HPPR*, *RAS* and *CYP98A14* (Figure 6).

The results showed that expression levels of genes in the upstream pathway (*PAL*, *C4H*, *4CL*, *TAT* and *HPPR*) were greatly promoted by Ag^+^ and reached their maximum on day 1 post-treatment. Their expression levels were 29.18-fold, 5.43-fold, 12.76-fold, 13.15-fold and 22.83-fold of the control levels, respectively. Then, expressions of those genes decreased gradually (Figure 6). This indicated *PAL* was more sensitive to Ag^+^ than *C4H*, *4CL*, *TAT* and *HPPR*. However, responses of two genes in the downstream pathway (*RAS* and *CYP98A14*) were later and reached to their maximum expression levels on day 6 after treatment, 16.14-fold (*RAS*) and 1.44-fold (*CYP98A14*) of the control levels, respectively (Figure 6). The results indicated that the upstream genes responded to Ag^+^ earlier than the downstream genes.

**Figure 7:**
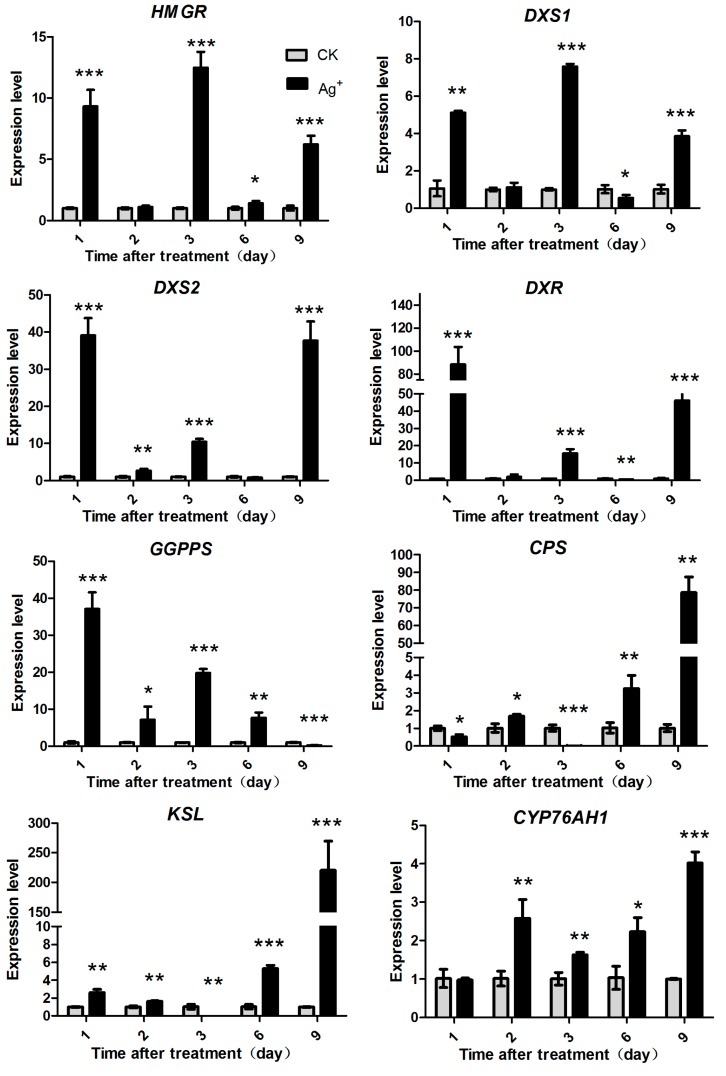
Effects of Ag^+^ on the expression level of seven key enzyme genes of RA tanshinones biosynthesis pathway (MEP pathway and MVA pathway) in *S. miltiorrhiza* hairy roots. The vertical bars show the SD values (*n* = 3). The asterisks indicate statistically significant differences at *p* < 0.05 (*, 0.01 < *p* <0.05; **, 0.001 < *p* < 0.01; ***, *p* < 0.001.) between the content in the elicitor treated cultures and that in the corresponding controls.

### 2.5. Effects of Ag^+^ Elicitor on Genes Expression Involved in Pathways of Tanshinones Biosynthesis

*HMGR*, *DXS1*, *DXS2*, *DXR*, *GGPPS* were key genes in the upstream pathways of tanshinones biosynthesis. *CPS*, *KSL* and *CYP76AH1* were key genes in the downstream pathways. In this study, expression levels of these genes were detected by RT-qPCR. The results showed that expressions of *HMGR*, *DXS1*, *DXS2* and *DXR* were dramatically induced by Ag^+^ on day 1 (Figure 7). Then their expressions decreased to control levels on day 2 and 6, but sharply increased on day 3 and 9. HMGR gene expression level increased by 9.32-fold, while *DXS1*, *DXS2*, *DXR* and *GGPPS* increased by 5.12-fold, 39.13-fold, 88.38-fold and 37.10-fold on day 1 post-treatment, respectively. However, *CPS*, *KSL* and *CYP76AH1* reached their maximum expression levels until day 9 after treatment, and increased by 78.67-fold, 220.50-fold and 4.02-fold of the control level, respectively. The results indicated that genes in the upstream pathway responded to Ag^+^ earlier than the downstream genes.

### 2.6. Discussion

Phenolics and tanshinones are two important groups of bioactive compounds in *S. miltiorrhiza*. The biosynthetic mechanisms of these compounds has been widely studied because of their medicinal value. Phenolic acids were mainly produced through the phenylpropanoid and tyrosine pathways, and tanshinones are biosynthesized via the MVA and MEP pathways. Various elicitors have been used to improve the accumulation of these bioactive ingredients in *S. miltiorrhiza* [31]. However, the regulation mechanisms of phenolic acid and tanshinone biosynthesis by elicitors is still unclear.

Among abiotic elicitors, heavy metals have a significant impact on plant growth and secondary metabolism. It was reported that Ca^2+^, Ag^+^, and Cd^2+^ could improve the production of tropane alkaloids in hairy roots cultures of *Brugmansia candida* [32]. Many kinds of heavy metal were also used as elicitors to induce accumulations of bioactive compounds in *S. miltiorrhiza*, such as Co^2+^, Ag^+^, Cd^2+^, Cu^2+^, Ce^3+^, La, Mn^2+^ and Zn^2+^ *etc.*, [12,21,24,33]. Among them, Ag^+^ was considered as an effective elicitor for phenolic compound and tanshinone production in *S. miltiorrhiza* hairy roots, and could improve RA, LAB and tanshinones production. But it was also reported that RA accumulation was almost not affected by Ag^+^ [29], so whether Ag^+^ have significant effect on phenolic acids remains unclear. No report compares how the two groups of bioactive compounds respond to Ag^+^.

LAB was considered as a derivative of RA, and its accumulation was affected by RA content [9]. In this study, we found that accumulations of all the six phenolic compounds were influenced by Ag^+^ (Figure 4). RA content reached 1.57-fold of the control (on day 9), but LAB content was reduced to 70% of the control. This result was the same as Yan’s report [12]. DSU was derived from 4-hydroxphenyllactic acid, and is a main degradation product of LAB [34]. In this work, we found that DSU accumulation was decreased by Ag^+^ as well as LAB. In another experiment of our lab, overexpression of RAS resulted in a decrease of LAB content, but increased RA content (unpublished data). Therefore, we speculated that DSU was probably a precursor of LAB biosynthesis and LAB was probably not biosynthesized from RA pathway but from DSU branch pathway (Figure 2). However, Xiao found that LAB was dramatically induced by Ag^+^ while RA content was not affected [29]. The reason of inconsistency may be the difference between bacterial strains used to obtain the hairy roots and the different harvest time we selected. Caffeic acid, ferulic acid and cinnamic acid are three upstream metabolites in the phenyl-propanoid pathway. Cinnamic acid is a precursor of caffeic acid and ferulic acid biosynthesis. Cinnamic acid accumulation was decreased, while accumulations of the two derivatives (caffeic acid and ferulic acid) were increased by Ag^+^. However, content of total phenolic acids was almost unaffected by Ag^+^ (Figure 5). It was probably because that metabolic flux among various phenolic acids was influenced by Ag^+^.

Changes of the six phenolic acids’ accumulations may be caused by gene expression in the phenylpropanoid and tyrosine pathways. PAL is the first key enzyme in the phenylpropanoid pathway, and its expression is closely related to accumulations of RA and other phenolics. Down-regulation of *PAL* markedly reduced accumulations of RA and LAB [35]. *C4H*, *TAT* and *HPPR* expressions were also related to accumulations of RA and LAB [36]. Our gene expression results indicated that both the phenylpropanoid and tyrosine pathways were affected by Ag^+^. Gene expressions in the upstream pathway responded to Ag^+^ earlier than those in the downstream pathway (Figure 7). This demonstrated Ag^+^ probably induced the whole phenolic acid biosynthesis pathway, and up-regulated gene expression from the upstream pathway to the downstream pathway, and this finally resulted in an enhancement of phenolic acid production.

As another group of bioactive compounds in *S. miltiorrhiza*, tanshinone production was also promoted by various elicitors (YE, fungi, PEG and ABA *etc.*) [10,33,37]. Ag^+^ has been widely reported to enhance tanshinone production in *S. miltiorrhiza* hairy roots [22,24,38]. Ge used 30 µM Ag^+^ to treat *S. miltiorrhiza* hairy roots, and found that the maximum content of the total tanshinones was 1.2-fold of the control [30]. In another report, 25 µM Ag^+^ could improve CT, T-I and T-IIA contents by 30-fold, 0.87-fold, 3.9-fold, respectively [33]. In this study, accumulations of four tanshinones components were also induced by Ag^+^ (Figure 5), and the maximum contents of T-I, T-IIA, CT and DT-I was 1.45-fold, 1.63-fold, 4.4-fold and 2.55-fold of the control, respectively. The maximum content of the total tanshinones was increased by 2-fold, which was much higher than the present reports. This indicated that Ag^+^ was an effective elicitor of tanshinone production, and CT was more sensitive to Ag^+^ than other tanshinone compounds.

The increase of tanshinone accumulation probably resulted from up-regulation of gene expression in the MEP and MVA pathways. With 30 µM Ag^+^ treatment, expression of many genes such as *HMGR*, *DXS2*, *GGPPS* and *CPS* in *S. miltiorrhiza* hairy roots was induced [22,39]. Our results showed that key genes in the two pathways were all induced by Ag^+^ (Figure 7). Expression of *HMGR* was increased by 9.31-fold (day 1) and expressions of *DXS1*, *DXS2*, *DXR* increased by 5.12-fold, 39.13-fold, 88.38-fold and 37.10-fold, respectively (day 1). It was indicated that the MEP pathway was more sensitive to Ag^+^ than the MVA pathway. This result was similar to previous reports [18]. However, expression of genes in the downstream pathway responded later than the upstream genes. The results were the same as those of the genes involved in phenolic acid biosynthesis.

## 3. Experimental Section

### 3.1. Hairy Roots Culture

*Agrobacterium rhizogenes* (ATCC15834) with Ri (root inducing) T-DNA (transfer DNA) was used to infect *S. miltiorrhiza* aseptic leaves, then the hairy roots of *S. miltiorrhiza* were obtained. 6,7-V liquid medium (with 30 g·L^−1^ sucrose) was used to culture the hairy roots. The hairy roots were maintained in a 100 mL beaker flask with 50 mL 6,7-V liquid medium. Each beaker flask was inoculated with 0.2 g fresh hairy roots and placed on an orbital shaker at 110 rpm·min^−1^, 25 °C in the dark. Elicitation was performed on the 18th day after inoculation. The hairy roots were harvested from the culture medium on days 1, 2, 3, 6 and 9 post-treatment. The fresh weight of each sample was recorded. Take half of each sample to isolate RNA, while the others were dried at 50 °C in an oven until constant dry weight for metabolites content analysis. Untreated hairy roots were designated as the control. The experiment was performed in triplicate, and the results are means ± SD.

### 3.2. Preparation of Ag^+^ Elicitor

Silver ions (Ag^+^) were supplied to the culture at a final concentration of 15 μM, using a concentrated silver thiosulfate (Ag_2_S_2_O_3_) solution prepared by mixing AgNO_3_ and Na_2_S_2_O_3_ at a molar ratio of 1:4. Then it was sterilized by filtration (0.22 μm membrane).

### 3.3. Metabolite Extraction and HPLC Analysis

Compound extraction and analysis followed the methods described by Yang [10] with minor modifications. The dried hairy root was ground to a powder with a mortar and pestle. The sample powder (50 mg) was extracted with 70% methanol (5 mL) under sonication for 45 min, then centrifuged at 8000 rpm (6010× *g*) for 10 min. The supernatant was ready to use for HPLC analysis. The metabolite contents were determined on a Waters HPLC system (Waters, Milford, MA, USA) equipped with a 1525 binary pump, a manual sample injector and a Waters 2996 photodiode array detector (PDA). Chromatography separation was performed with a SunFire C18 column (4.6 mm × 250 mm, 5 mm particle size) at 30 °C. Empower 2 software was used for data acquisition and processing. The sample injection volume was 20 µL and the PDA detection wavelength for the lipid-soluble diterpenoids were 270 nm, and for the water-soluble phenolic acids was 280 nm. Separation was achieved by elution using a linear gradient with solvent-A (acetonitrile) and solvent-B (0.02% phosphoric acid solution). The gradient was as follows: *t* = 0 min, 5% A; *t* = 10 min, 20% A; *t* = 15 min, 25% A; *t* = 20 min, 25% A; *t* = 25 min, 20% A; *t* = 28 min, 30% A; *t* = 36 min, 30% A. The flow rate was 1.0 mL·min^−1^. Standards of metabolite compounds were purchased from the National Institute for the Control of Pharmaceutical and Biological Products (Beijing, China) [8].

### 3.4. RNA Isolation and Real-Time Quantitative PCR Analysis

Total RNAs from *S. miltiorrhiza* hairy roots were extracted at selected time points using RNAiso^TM^ Plus (Takara, Tokyo, Japan) according to the manufacturer’s instructions. The quality and concentration of RNA were examined by ethidium bromide (EB)-stained agarose gel electrophoresis and spectrophotometer analysis (Thermo Scientific NanoDrop 2000). The first strand cDNA for RT-qPCR was synthesized from 1 µg total RNA using the PrimeScript™ RT reagent Kit with gDNA Eraser (Perfect Real Time, Takara, Tokyo, Japan). Primers used for RT-qPCR were list at Table 1. The constitutively expressed 18S *rRNA* with specific primers F18S (5’-ATGATAACTCGACGGATCGC-3’) and R18S (5’-CTTGGATGTGGTAGCCGTTT-3’) were used as control. Real-time PCR was performed according to the manufacturer’s instruction of SYBR^®^ Premix Ex Taq™ II (TliRNaseH Plus, Takara) using the following protocol: 95 °C, 30 s, 1 cycle; 95 °C, 5 s, annealing temperature (Tm), 30 s, 40 cycles. The program was performed on the Mastercyclerep realplex4 system (Eppendorf, Hamburg, Germany). Quantification of the gene expression was done with comparative CT method (2^−ΔΔCT^). Experiments were performed in triplicate, and the results were represented by their means ± SD.

**Table 1:** List of RT-qPCR primers.

Gene	Sense Primer (5’ to 3’)	Reverse Prime (5’ to 3’)	Genbank ID
**PAL**	GGCGGCGATTGAGAGCAGGA	ATCAGCAGATAGGAAGAGGAGCACC	GQ249111
**4CL**	TCGCCAAATACGACCTTTCC	TGCTTCAGTCATCCCATACCC	AY237164
**C4H**	CCAGGAGTCCAAATAACAGAGCC	GAGCCACCAAGCGTTCACCAA	EF377337
**TAT**	TTCAACGGCTACGCTCCAACT	AAACGGACAATGCTATCTCAAT	DQ334606
**HPPR**	GACTCCAGAAACAACCCACATT	CCCAGACGACCCTCCACAAGA	DQ099741
**CYP98A14**	CTAAGGAGGTGCTGAAGGAG	GTGGAGTCGTTGTAGATGGA	HQ316179
**RAS**	CGCCCTAGTTGAGTTCTACCCTTACGC	TCGGATAGGTGGTGCTCGTTTGC	FJ906696
**HMGR**	GCAACATCGTCTCCGCCGTCTACA	GATGGTGGCCAGCAGCCTGGAGTT	FJ747636
**DXS1**	CGACCAGGTAGTGCACGACG	TCATCTGAAGGAGCCATCACCAC	EU670744
**DXS2**	TTGGAGATTGGGAAGGGAAGGAT	AGGCTTGCAGAATCTCGCATCAG	FJ643618
**DXR**	GAGAATCTACTGCTCCGAGA	CTGGTCGTAGTGGATGATCT	DQ991431
**GGPPS**	GGGGCTATTTTGGGAGGTGGAA	CAGCAGCTTGGGATACGTGGTC	FJ178784
**CPS**	GAGGGAGAGGTGAGGAAGGAA	AGGGAACAAAAGTTGAAAAGG	EU003997
**KSL**	CATGTCGAACAAGGACGTA	AATCATCCAAGGTTAGTGCC	EF635966
**CYP76AH1**	CAGGAGGTGAACGGCTATCT	GTTATGAACCAGAGTCGCAGTAG	JX422213

## 4. Conclusions

In conclusion, the production of both phenolic acids and tanshinones except DSU, LAB and cinnamic acid were enhanced by Ag^+^ treatment, but Ag^+^ was more effective at stimulating tanshinone production than that of phenolic acids. LAB was probably derived from the branch pathway of DSU biosynthesis, and the MEP pathway was more sensitive to Ag^+^ than the MVA pathway. Genes in the upstream pathways responded to Ag^+^ earlier than those in the downstream pathway. Ag^+^ probably induced the whole pathways of those ingredients’ biosyntheses, upregulated gene expressions from the upstream pathway to the downstream pathway, and this finally resulted in the enhancement of ingredient production. This study will help us understand how secondary metabolism in *S. miltiorrhiza* responds to elicitors and provide a reference for the improvement of targeted compound production in the near future.

## Abbreviation

ABA: abscisic acid
C4H: cinnamic acid 4-hydroxylase
CPS: copalyl diphosphate synthase
CT: cryptotanshinone
CYP98A14: cytochrome P450-dependent monooxygenase
CYP76AH1: cytochrome P450-dependent monooxygenase
DMAPP: dimethylallyl diphosphate
DTI I: dihydrotanshinone I
DXR: 1-deoxy-d-xylulose 5-phosphate reductoisomerase
DXS: 1-deoxy-d-xylulose 5-phosphate synthase
DW: dry weight
EB: ethidium bromide
FW: fresh weight
GA: gibberellins acid
GGPP: geranylgeranyl diphosphate
GGPPS: Geranylgeranyl diphosphate synthase
HMGR: 3-hydroxy-3-methylglutaryl CoA reductase
HPLC: high performance liquid chromatography
HPPR: 4-hydroxyphenylpyruvate reductase
IPP: isopentenl diphosphate
KSL: kaurene synthase-like
LAB: salvianolic acid B
MeJA: methyl jasmonate
MEP: methylerythritol phosphate
MVA: mevalonate
PAL: Phenylalanine ammonia-lyase
PDA: photodiode array detector
PEG: polyethylene glycol
RA: rosmarinic acid
RAS: rosmarinic acid synthase
RNAi: RNA interference
RT-qPCR: quantitative real-time PCR
SA: salicylic acid
*Salvia miltiorrhiza*: *Salvia miltiorrhiza*
TAT: tyrosine aminotransferase
T I: tanshinone I
T II A: tanshinone II A
YE: yeast extract
4CL: hydroxycinnamate coenzyme A ligase
